# The effects of weight fluctuation on the components of metabolic syndrome: a 16-year prospective cohort study in South Korea

**DOI:** 10.1186/s13690-021-00539-x

**Published:** 2021-02-18

**Authors:** Young Ran Chin, Eun Sun So

**Affiliations:** 1 Department of Nursing, Chungwoon University, 25 Daehak-gil, Hongseong-eup, Hongseong, 32244 Republic of Korea; 2 College of Nursing, Jeonbuk National University, 567 Baekje-daero, Deokjin-gu, Jeonju-si, Jeollabuk-do 54896 Republic of Korea

**Keywords:** Weight fluctuation, Metabolic syndrome, Abdominal obesity

## Abstract

**Background:**

Weight fluctuation (WF) is highly prevalent in parallel with the high prevalence of intentional or unintentional dieting. The health risks of frequent WF for metabolic syndrome (MS) have become a public health concern, especially for health care providers who supervise dieting as an intervention to prevent obesity-related morbidity or to improve health, as well as for the general population for whom dieting is of interest. The aim of this study was to investigate the long-term effect of WF on the risk of MS in Koreans.

**Methods:**

This study analyzed secondary data from the Korean Genome and Epidemiology Study, a 16-year prospective cohort study, on 8150 individuals using time-dependent Cox regression.

**Results:**

WF did not increase the risk of MS in either normal-weight or obese subjects. In an analysis of the components of MS, greater WF significantly increased the risk of abdominal obesity (HR = 1.05, 95% CI = 1.02–1.07, *p* < 0.001) in normal-weight individuals. However, WF did not increase the risk of hyperglycemia, low high-density lipoprotein cholesterol levels, elevated blood pressure, or raised fasting glucose in normal-weight individuals, and it did not influence any of the components of MS in obese individuals.

**Conclusion:**

Since WF was found to be a risk factor for abdominal obesity, which is the most reliable predictor of MS, it should be considered when addressing weight control. Further studies on cut-off points for the degree of weight loss in a certain period need to be conducted to help clinicians provide guidance on appropriate weight control.

## Background

Dieting, which refers to weight control behaviors with the intention to lose weight or fat [1], is highly prevalent worldwide regardless of individuals’ obesity status [1, 2]. Most people who are obese or overweight go on a diet on one or more occasions in their lifetime given the health risks and discrimination associated with obesity, and those who are non-obese often diet due to socio-cultural pressures to attain a slim figure, as well as in response to concerns regarding the emergence of an obesogenic environment [1, 2].

In parallel to the prevalence of dieting, weight fluctuation (WF)—also commonly known as frequent weight change or weight cycling—is also highly prevalent and is also not restricted to those who are obese or overweight [1, 2]. The health risks of frequent WF have been emphasized as a public health concern, especially for health care providers who supervise dieting as an intervention to prevent obesity-related morbidity or to improve health, as well as for the general population for whom dieting is of interest [2–4]. This is because WF and MS are known to increase morbidity and mortality due to cardiovascular disease, an important cause of human death worldwide [5–8].

In South Korea, the age-adjusted prevalence of metabolic syndrome has significantly increased from 24.9% in 1998, 29.2% in 2001, and 30.4% in 2005 to 31.3% in 2007. Abdominal obesity and dyslipidemia were identified as major contributing factors to the increased prevalence of metabolic syndrome in Korea during this 10-year period [9]. Therefore, extensive research has been conducted through the Korean Genome and Epidemiology Study (KoGES) with a focus on metabolic syndrome and T2DM because of the relatively high prevalence of these conditions in the population and clear disease ascertainment [10].

Although some studies have attempted to provide evidence for the common perception that WF increases cardiovascular disease [5–8], the findings are inconsistent and sparse [1, 11, 12]. Various interpretations have been put forth regarding the potential association between WF and cardiovascular disease. One explanation is that WF is associated with metabolic disturbances, such as insulin resistance [13], abdominal fat accumulation [14, 15], and an increased risk for diabetes [6, 16]. Another explanation is that people with risk factors for CVD are more likely to have WF as a result of their daily behaviors. However, the above explanations are assumptions with no clear evidence [8].

Previous review studies on this topic have mostly shown non-deleterious associations with WF or have only found deleterious effects in non-obese populations [1, 11]. Metabolic syndrome (MS), a cluster of cardiovascular risk factors including obesity, insulin resistance, dyslipidemia, and hypertension, is associated with elevated risks for type 2 diabetes and cardiovascular disease [17, 18].

Therefore, this study was conducted to investigate the long-term contribution of body-weight variability to MS after adjusting for well-known risk factors in community-dwelling adults in South Korea.

## Methods

### Study data and quality control

This study used secondary prospective cohort data from the KoGES (Korean Genome and Epidemiology Study) [10], conducted by the National Institute of Health of the Korean Centers for Disease Control and Prevention (KCDC). The KoGES was conducted to establish a genomic and epidemiologic database for examining genetic and environmental effects on the prevalence of major noncommunicable diseases, such as diabetes mellitus, hypertension, obesity, and MS in Koreans.

In total, 10,030 adults aged 40–69 years living in two cities—Ansan (urban) or Ansung (rural)—in Korea were recruited at 28 health examination centers as the baseline cohort in 2001–2002 and were followed up biennially to November 2016. All participants completed interviewer-administered questionnaires on demographic information, lifestyle factors (including dietary habits), their health condition, and medical history, and anthropometric measurements were also acquired every 2 years and biochemical tests were conducted biennially.

Study participants recruited at 28 health examination centers across the country took part in questionnaire-based interviews conducted by trained interviewers to obtain their sociodemographic and health behavioral information, and anthropometric measurements and clinical examinations were performed by trained personnel based on the study’s protocol.

The detailed profile and methods of the construction of the KoGES cohort have been described elsewhere [10]. The KoGES participants were recruited with the cooperation of local organizations such as public health centers, and after completing a written consent form to participate in the study, they received questionnaires and checkups about their health status and lifestyle, which lasted for approximately 2–3 h. In order to reduce the inter- and intra- observer bias between the two cohorts in Anseong and Ansan, standardization training between and within cohorts was regularly conducted starting from the baseline survey. Standardized education and practice with Anseong and Ansan cohort researchers were conducted with a focus on training researchers to measure items with the potential for errors in test values, ​​such as questionnaire methods, blood pressure measurement techniques, diabetes test methods, and standardization of device use. No significant differences were found among the measured values. In addition, within the cohort, maintenance training was regularly conducted three to four times each year.

The questionnaire data were generated through two steps, in which a surveyor interviewed a participant using a questionnaire and then entered the content into the system. To prevent input errors, the questionnaire results were entered twice and data were saved only if the first and second input results matched.

Over the course of the study, promotional materials were regularly sent to each participant, and several phone calls were made to confirm health information, disease status, death, and cause of death after baseline/tracking investigations through the person and his/her family. In particular, the death information from the National Statistical Office was used as secondary data, in addition to the data collected through the cohort, to reconfirm the occurrence of death, the date of death, and the cause of death.

All collected data were input and managed in the KoGES program of the Cohort Epidemiologic Information System by the Korean National Institute of Health. This process was thoroughly managed through the following separate processes (1) integration of collected data, (2) initial quality control, (3) data cleaning for single variables, (4) data cleaning for related variables, and (5) statistical quality control.

### Study subjects

**Fig. 1:**
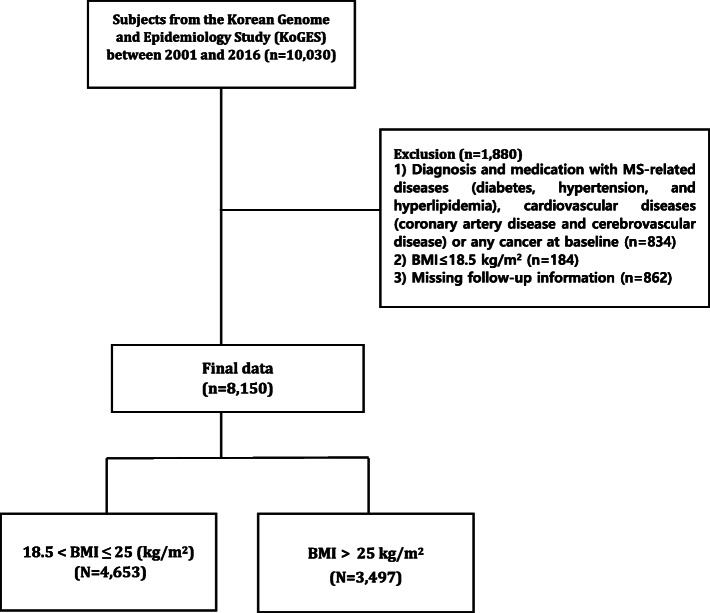
Flowchart of study population: a 16-year prospective prospective cohort study in South Korea

Of these 10,030 subjects, 8150 individuals were analyzed after excluding those (*n* = 1880) who 1) were diagnosed with and prescribed medications for MS-related diseases (diabetes, hypertension, and hyperlipidemia), cardiovascular diseases (coronary artery disease and cerebrovascular disease), or any cancer at baseline (*n* = 834); 2) were underweight (*n* = 184); or 3) missed a follow-up survey (*n* = 862). Subjects with MS-related diseases, cardiovascular diseases, or cancer were excluded to determine more precisely the association between WF and incident MS or its components and to exclude patients with factors known to be associated with unintentional weight loss [20]. Underweight participants were excluded because they comprised an insufficient sample size. We classified the participants into two groups—non-obese and obese—according to their obesity status in the final analysis (Fig. 1).

### Ethical considerations

The KoGES study protocol was reviewed and approved by the Institutional Review Board of the KCDC, and all study participants submitted written informed consent before enrollment.

Participants voluntarily completed a self-administered questionnaire, which included questions on their previous medical history and health-related behavior. Anthropometric measurements and laboratory tests were conducted as part of a general health check-up, and participants were informed of the results.

This research was approved by the institutional review board of Jeonbuk National University (JBNU 201803019). The KCDC provided the raw data after reviewing our study’s plan and IRB approval.

### Data sharing

We sent our research plan and IRB approval from our affiliated institution to the KCDC, and pledged to use the data appropriately. After that, the raw data were downloaded from the website.

### Measurements

#### Independent variables

The independent variables included WF, with covariates including age at baseline, sex, education (elementary school, middle school, high school, junior college, or university graduation), marital status (divorced, widowed, single/married, or cohabiting), monthly income (10,000 Korean won), drinking status, and smoking status. The health behavioral information on drinking (never drinker in one’s lifetime, ex-drinker, or current drinker) and smoking status (never smoker in one’s lifetime, ex-smoker, or current smoker) was self-reported [10].

WF was calculated using weight at each follow-up using the following formula: (|weight_1_- weight_2_| + |weight_2_- weight_3_| + ... + |weight_n-1_-weight_n_|)/n-1. We calculated WF including the body-weight values from baseline to just before the event (MS) was captured. WF was defined as the sum of the absolute values ​​of the previous weight minus the next weight divided by the number of measurements minus 1. In other words, from 2001 to 2002 to 2016, a maximum of 8 measurements were performed every 2 years, and subjects who missed more than 3 examinations were excluded, so the sum of at least 4 absolute values of weight differences were divided by 3 or more. A larger WF means a larger weight change during the cohort data collection period.

The body weight and height were measured to evaluate participants’ level of obesity in terms of the body mass index (BMI). Body weight and height were measured to the nearest 0.1 cm and 0.1 kg, respectively, while participants wore light clothes without shoes. BMI was calculated as kilograms per meter squared, and normal-weight and obese subjects were classified as 18–25 kg/m^2^ and > 25 kg/m^2^ respectively, in keeping with the cut-off values recommended for Asian populations [7, 10]. The criteria described are widely used in Korea and Japan to compensate for the problem that the universal standard proposed by the World Health Organization [21] is not suitable for Asians [22, 23]. There are no existing findings regarding body-weight fluctuation and mortality in Asian populations [7], and to the best of our knowledge, there is no standard of obesity generally recognized to be suitable for Asians.

#### Dependent variables

The dependent variables included MS and its five components. MS was defined by the presence of three or more of the following five components according the Adult Treatment Panel using waist circumference for Asians [24]: 1) central obesity (≥ 90 cm for men or ≥ 80 cm for women), 2) low high-density lipoprotein cholesterol (HDL-C) (< 40 mg/dL for men or < 50 mg/dL for women), 3) hypertriglyceridemia (a triglyceride level ≥ 150 mg/dL), 4) elevated blood pressure (BP) as defined by a systolic BP ≥ 130 mmHg or a diastolic BP ≥ 85 mmHg, and 5) raised fasting glucose (≥100 mg/dL).

Waist circumference was measured midway between the inferior margin of the last rib and the crest of the ilium in a horizontal plane with units of 0.1 cm. Blood pressure was measured twice on the subject’s dominant arm while in the sitting position with a 5-min interval between readings using a mercury sphygmomanometer, and the mean of the two measurements was reported.

A blood sample for triglycerides, fasting glucose, and HDL-C was drawn after 12 h of fasting and laboratory tests were performed using a Hitachi 747 chemistry analyzer at a central laboratory.

### Data analysis

Continuous variables were reported as mean ± standard deviation and categorical variables were expressed as numbers and percentages to describe the sociodemographic and health behavioral characteristics of the study subjects. Comparisons between the normal BMI group and obese BMI group were performed using the Student t-test for continuous variables, and the chi-square test for categorical variables. Kaplan-Meier curves were used to calculate cumulative MS incidence rates according to obesity status, and the statistical significance of differences was compared using the log-rank test. Since people’s weight changes over time, time-dependent Cox regression was performed to reflect changes in weight during follow-up on the risk of MS and its components [25]. The time segments corresponded to weight measurements at intervals of 2 years or more. The models were adjusted for sociodemographic and health behavioral variables. The results are presented as hazard ratios (HRs) and 95% confidence interval (CIs). All reported *P*-values are two-sided, and P-values < 0.05 were considered to indicate statistical significance. All analyses were performed with SAS software, version 9.4 (SAS Institute, Cary, NC, USA).

## Results

The baseline sociodemographic, health behavioral, and physiological data of the study subjects are shown in Tables 1 and 2. Of a total of 8150 subjects, 57.1% (4653 individuals) had a normal BMI (18–25 kg/m^2^), and 42.9% (3497 individuals) had an obese BMI (> 25 kg/m^2^). Overall (i.e., in both groups), the participants were on average 51 years old and were predominantly female (53.2%), less educated (32.7% for elementary education), married/cohabiting (90.6%), lower-income (earning less than 2000,000 Korean won monthly, or about 2000 dollars) (48.4%), current drinkers (48.1%), and never smokers (49.6%). The obese group showed statistically significantly higher proportions of female subjects (55.9% vs. 50.4%), subjects with a lower education level (34.4% vs. 30.4% for elementary education), and subjects with a higher monthly income (8.7% vs. 6.9% for earning more than 4,000,000 Korean won), and never and ex- drinkers and smokers (53.6% vs. 50.3 and 78.0% vs. 71.9%) than the normal-weight group. No significant differences in marital status or age between groups were observed. The normal and obese groups had mean BMIs of 22.60 kg/m^2^ and 27.43 kg/m^2^ and mean weights of 58.32 kg and 69.91 kg, respectively. The mean WF ​​during the cohort period was 1.71 ± 4.6 kg in the normal BMI group and 2.04 ± 5.73 kg in the obese BMI group.

**Table 1:** Sociodemographic and health behavioral characteristics at baseline: a 16-year prospective prospective cohort study in South Korea (n, %)

		18 < BMI ≤ 25 (kg/m^2^) (*n* = 4653, 57.1%)	BMI > 25 kg/m^2^ (*n* = 3497, 42.9%)	*P*-value
Age at baseline (years)	(mean ± SD)	51.83 ± 9.03	51.60 ± 8.41	0.247
	40–49	2313(49.7)	1733(49.6)	
	50–59	1141 (24.5)	978 (28.0)	
	60–69	1199 (25.8)	786 (22.5)	
Sex	Male	2310 (49.6)	1542 (44.1)	< 0.001
	Female	2343 (50.4)	1955 (55.9)	
Education	Elementary school	1425 (30.9)	1193 (34.4)	0.001
	Middle school	1071 (23.2)	798 (23.0)	
	High school	1506 (32.6)	1001 (28.9)	
	Junior college	179 (3.9)	123 (3.5)	
	University	431 (9.4)	335 (9.6)	
Marital status	Divorced/widowed/single	423 (9.2)	335 (9.6)	0.455
	Married/cohabiting	4199 (90.8)	3140 (90.4)	
Monthly income (10,000 won)	< 100	1609 (35.2)	1110 (32.4)	0.007
	100–200	1333 (29.2)	1039 (30.3)	
	200–300	855 (18.7)	631 (18.4)	
	300–400	457 (10.0)	351 (10.2)	
	≥400	315 (6.9)	298 (8.7)	
Drinking	Never drinker	2049 (44.5)**	1636 (47.2)	0.010
	Ex-drinker	266 (5.8)	211 (6.4)	
	Current drinker	2294 (49.7)	1607 (46.4)	
Smoking	Never smoker	2621 (57.0)***	2141 (62.2)	< 0.001
	Ex-smoker	685 (14.9)	545 (15.8)	
	Current smoker	1291 (28.1)	759 (22.0)	

*BMI* Body mass index

**Table 2:** MS characteristics at baseline and weight fluctuation: a 16-year prospective cohort study in South Korea (mean ± SD)

	18 < BMI ≤ 25	BMI > 25 kg/m^2^	
	*n* = 4653 (57.1%)	*n* = 3497 (42.9%)	*P-*value
BMI (kg/m^2^)	22.60 ± 1.62	27.43 ± 2.08	< 0.001
Weight (kg)	58.32 ± 7.42	69.91 ± 8.88	< 0.001
Height (cm)	160.41 ± 8.45	159.49 ± 8.92	< 0.001
Weight fluctuation (kg)	1.71 ± 4.61	2.04 ± 5.73	
Waist circumstance (cm)	78.30 ± 6.71	88.75 ± 7.06	< 0.001
HDL-C level (mg/dL)	51.55 ± 12.09	46.90 ± 10.58	< 0.001
Triglyceride levels (mg/dL)	135.00 ± 95.28	173.30 ± 117.10	< 0.001
BP (systolic BP) (mmHg)	119.20 ± 18.26	125.10 ± 18.26	< 0.001
Fasting glucose (mg/dL)	89.25 ± 18.13	93.45 ± 19.96	< 0.001

*MS* Metabolic syndrome, *BMI* Body mass index, *HDL-C* High-density lipoprotein cholesterol, *BP* Blood pressure

**Fig. 2:**
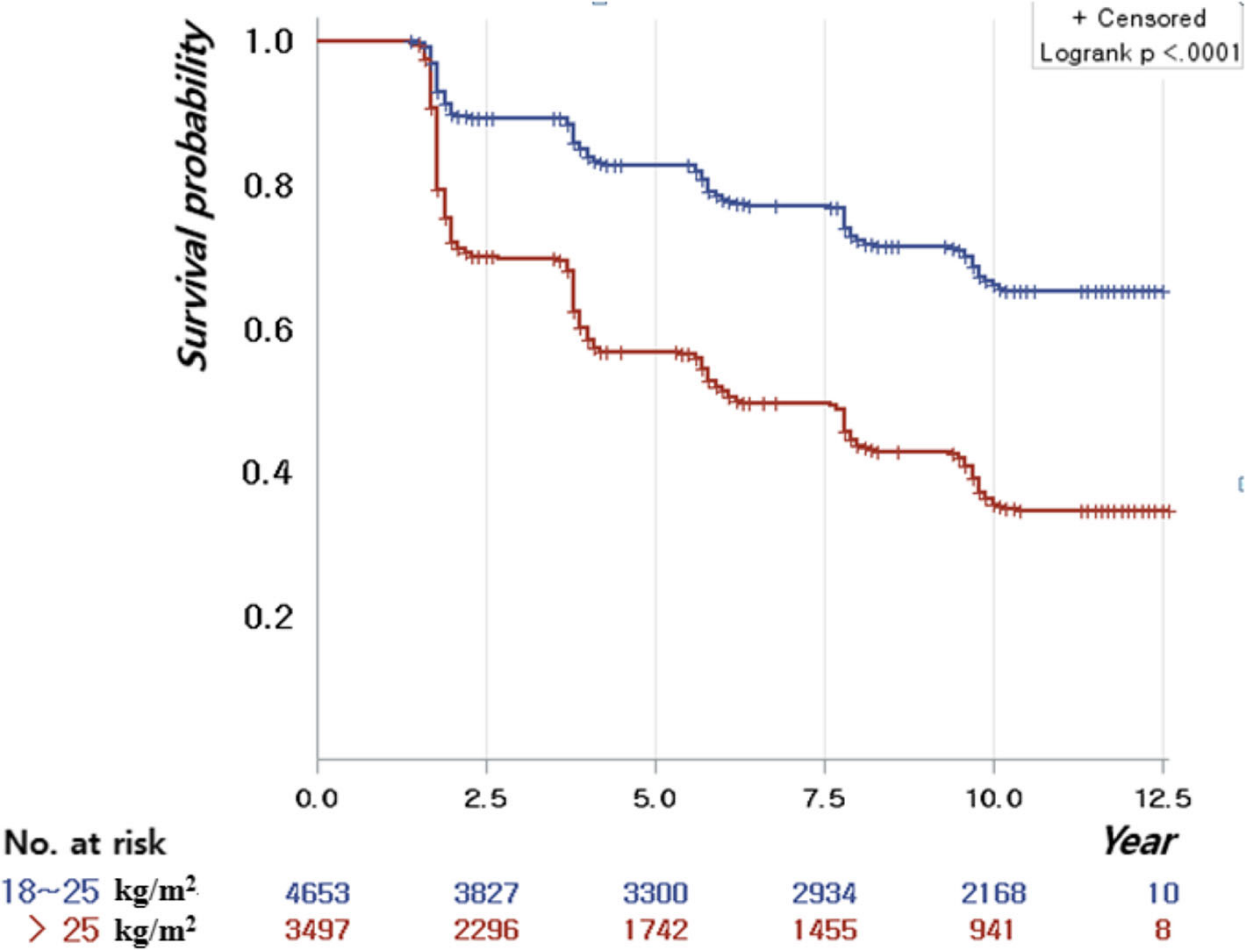
Kaplan–Meier curves for the risk of metabolic syndrome: a 16-year prospective prospective cohort study in South Korea

The Kaplan-Meier curves illustrate the trend of risk for MS according to BMI groups. MS showed a significantly higher incidence in the obese group than in the normal-weight group (log-rank test *p* < 0.001) (Fig. 2). As shown in Tables 3 and 4, time-dependent Cox proportional hazards models were constructed to investigate the association between WF and the risk of MS and its components. After adjusting for age, sex, education, marital status, monthly income, drinking status, and smoking status, a greater magnitude of WF did not increase the risk of incident MS, regardless of baseline obesity status. In addition, in the normal-weight group, older age at baseline, lower education, lower income, and current smoking were associated with a higher risk of MS. In the obese group, older age, male sex, lower education, lower income, and current smoking were associated with a higher risk of MS.

**Table 3:** Time-varying Cox proportional hazards models of weight change for metabolic syndrome: a 16-year prospective cohort study in South Korea

		18 < BMI ≤ 25 (kg/m^**2**^)		BMI > 25 kg/m^**2**^	
		Adjusted HR (95% CI)	*P*-value	Adjusted HR (95% CI)	*P*-value
**WF**		1.01 (0.98–1.04)	0.569	1.00 (0.98–1.02)	0.726
**Age at baseline**		1.04 (1.03–1.04)	< 0.001	1.02 (1.01–1.02)	< 0.001
**Sex**	Male	1		1	
	Female	1.11 (0.92–1.34)	0.266	0.83 (0.71–0.97)	0.021
**Education**	Elementary school (from 1st to 6th grade)	1		1	
	Middle school (from 7th to 9th grade)	0.88 (0.75–1.03)	0.100	0.80 (0.70–0.91)	0.001
	High school (from 10th to 11th grade)	0.69 (0.59–0.82)	< 0.001	0.75 (0.65–0.86)	< 0.001
	Junior college	0.67 (0.47–0.95)	0.023	0.69 (0.52–0.91)	0.014
	University	0.63 (0.50–0.81)	0.003	0.64 (0.53–0.78)	< 0.001
**Marital status**	Divorced/widowed/single	1		1	
	Married/cohabiting	0.86 (0.72–1.03)	0.028	0.98 (0.84–1.15)	0.833
**Monthly income (10,000 won)**	< 100	1		1	
	100–200	0.72 (0.62–0.83)	< 0.001	0.87 (0.77–0.98)	0.019
	200–300	0.63 (0.52–0.76)	< 0.001	0.68 (0.59–0.8)	< 0.001
	300–400	0.60 (0.48–0.76)	< 0.001	0.59 (0.49–0.72)	< 0.001
	≥400	0.59 (0.45–0.77)	0.001	0.61 (0.5–0.74)	< 0.001
**Drinking**	Never drinker	1		1	
	Ex-drinker	0.98 (0.76–1.25)	0.857	0.97 (0.8–1.18)	0.746
	Current drinker	0.95 (0.83–1.09)	0.435	0.93 (0.83–1.04)	0.177
**Smoking**	Never smoker	1		1	
	Ex-smoker	1.20 (0.97–1.49)	0.091	0.95 (0.80–1.13)	0.555
	Current smoker	1.77 (1.47–2.14)	< 0.001	1.33 (1.14–1.55)	0.003

The HR model was adjusted for age, sex, education, marital status, monthly income, drinking, and smoking

*HR* Hazard ratio, *CI* Confidence interval, *WF* Weight fluctuation, *BMI* Body mass index

**Table 4:** Time-varying Cox proportional hazards models of weight change for metabolic syndrome components after adjusting for covariates: a 16-year prospective cohort study in South Korea

	18 < BMI ≤ 25 (kg/m^**2**^)		BMI > 25 kg/m^**2**^	
	Adjusted HR (95% CI)	*P*-value	Adjusted HR (95% CI)	*P*-value
Abdominal obesity	1.05 (1.02–1.07)	< 0.001	1.01 (1.00–1.03)	0.349
Reduced HDL-C	0.99 (0.97–1.02)	0.349	1.01 (1.00–1.03)	0.372
Hypertriglyceridemia	0.99 (0.97–1.02)	0.588	0.99 (0.96–1.01)	0.819
Elevated BP	1.01 (0.98–1.04)	0.810	0.99 (0.97–1.02)	0.313
Raised fasting glucose	1.02 (1–1.03)	0.344	1.01 (1–1.03)	0.211

The HR model was adjusted for age, sex, education, marital status, monthly income, drinking, and smoking

*HR* Hazard ratio, *CI* Confidence interval, *BMI* Body mass index, *HDL-C* High-density lipoprotein cholesterol, *BP* Blood pressure

As for the association between WF and the risk of MS components, greater WF significantly increased only the risk of abdominal obesity (HR = 1.05, 95% CI = 1.02–1.07, *p* < 0.001) in the normal-weight group. It did not increase the risk of hyperglycemia, low HDL-C levels, elevated BP, or raised fasting glucose in the normal-weight group, and it did not influence any of the MS components in the obese group.

## Discussion

This study found that WF had no statistically significant effect of MS in either normal-weight or obese individuals. Furthermore, with regard to the components of MS, WF significantly increased only the risk of abdominal obesity and did not increase the risk of hyperglycemia, low HDL-C levels, elevated BP, and raised fasting glucose in the normal-weight group, and it did not influence any of the components of MS in the obese group. A multiethnic cohort study reported that Japanese American women had greater abdominal and visceral adiposity than Caucasian women with similar BMI [26]. The researchers suggested that even with the same BMI, management strategies should be more sensitive in Asian women, and that interventions should start at a lower BMI. Among Japanese men, long-term WF was associated with MS independent of current BMI [5]. The finding that only abdominal obesity was significant in our study could be considered in light of the finding of greater abdominal adiposity in Japanese American women, given the geographic proximity of South Korea to Japan.

The findings of previous studies on the relationship between WF and MS are sparse and inconsistent, which has made this relationship a topic of ongoing interest. Some previous studies suggested that WF increased the risks of MS [5, 27], while others did not [1, 2]. The following factors may explain the diversity of previous study findings: (1) a universally accepted definition of WF does not exist, so the measurements used in previous studies were diverse; (2) subjects were mostly classified qualitatively according to researchers’ arbitrary cut-off points, thereby inducing classification bias; and (3) variation in statistical methods, such as quantitatively estimating the slope, the trend index (regression coefficient), and the deviation from the slope, the WF index (the root mean square error), or defining WF as at least two episodes of weight variation (e.g., weight loss followed by weight regain) [5, 27]. The comparability of the results of the latter two studies is also limited due to differences in how WF was defined in terms of length, amplitude, and frequency [1, 11]. Intermittent weight measurements may conceal a number of unmeasured fluctuations in the interim, potentially underestimating the influence of WF on the risk of MS [1, 28]. At the same time, those studies counted consecutive weight loss or weight gain once, rather than separately, as weight change; since the amplitude of WF has a greater impact than its frequency on the risk of MS [12, 27], defining weight change in this way may have led to an overestimation of MS risk.

Although most studies related to WF have used the term WF interchangeably with weight change, weight cycling, and yo-yo dieting, the definitions of these terms should be clearly distinguished. Weight change means either an increase or decrease in weight, weight cycling and yo-yo dieting refer to intentional weight loss followed by unintentional weight regain [1, 11], and WF refers to successive episodes of weight change, especially continuous episodes of weight change in any direction, even including successive weight loss or weight gain. This definition of WF was applied in the present study, as distinct from the other similar definitions. Furthermore, to minimize the impact of making WF measurements at varying intervals, time-dependent Cox regression analysis was conducted in this study, because this technique is well suited to represent WF and helps avoid bias introduced by analyzing time course variables in combination with survival time [25, 29].

Regarding the associations of WF with MS components, this study found that WF increased the risk of abdominal obesity in the normal-weight group, but not in the obese group. This finding is consistent with previous findings that WF showed a valid relationship with MS in those who were lean, but that conflicting results were found in those who were obese [11, 17, 19]. This is reasonable because it is likely that obese individuals are more likely to already have abdominal obesity, thereby leading to an underestimation of the effects of WF on abdominal obesity in this group. Furthermore, it was demonstrated in this study that older age affected the risk of MS, in accordance with previous proposals that age-related changes, including weight gain after weight loss, increases in fat, and changes in the distribution of fat in the body (such as increased fat accumulation in the trunk in men and in the limbs in women), can be explained through both metabolic routes (e.g., the loss of lean mass, adaptive thermogenesis, reduced metabolic rate, and fat overshooting) and psychological routes (e.g., preoccupations with food and food obsession) [11, 17, 19, 30–32]. Since waist circumference has been identified to be a good predictor of various health outcomes, including MS [33, 34], and Asians (including Koreans) have more visceral fat tissue at specific levels of obesity than other races [34, 35], the effect of WF on abdominal obesity should be noted. However, in this study, associations were not found between WF and hyperglycemia, low HDL-C levels, elevated BP, or raised fasting glucose.

A limitation of this study is that weight was measured at quite a long interval (2 years or longer), which may make it difficult to identify episodes of WF in the meantime. This may have led to an underestimation of the risk of WF on the health outcomes analyzed in this study [1, 28]. However, a previous study demonstrated that the amplitude of WF was more closely associated with the risk of MS than its interval [27]. Furthermore, since the number of subjects was large and the measurement period for each subject was regular (roughly every 2 years), we expect that this limitation would have been slightly offset.

However, this study also has important strengths. First, a large and representative national Asian study population was used. Second, a longitudinal method, which is suitable for identifying cause-and-effect relationships [36], was used. Finally, we made a novel contribution by attempting to analyze WF based on a careful definition of WF that differentiated it from other similar terms. The definition of WF and the statistical methods deployed herein could be used in other studies.

We used BMI as an indicator of obesity. However, it has been proposed that waist circumference, as an indicator of fat distribution, may be a more accurate criterion for Asians. Therefore, future studies should also analyze waist circumference.

## Conclusion

In a 16-year cohort study, the relationship between WF and MS, which is a risk factor for cardiovascular disease, was not significant in either normal-weight or obese individuals. Only abdominal obesity in normal-weight individuals was affected by WF. Since WF slightly affected the abdominal obesity of normal-weight individuals, health care personnel should pay attention to WF when working towards weight control in patients with a normal weight. In other words, we should focus on whether patients are losing enough weight to maintain a healthy weight, rather than losing excessive weight. Furthermore, no single clear criterion is widely accepted for obesity or WF, so it may be necessary to repeat this research using other criteria than BMI.

## Data Availability

The datasets generated during the current study are available in the Korea National Health and Nutrition Examination Survey (KNHANES) repository, https://knhanes.cdc.go.kr/knhanes/eng/index.do

## Abbreviations

WF: Weight fluctuation
MS: Metabolic syndrome
HR: Hazard ratio
CI: Confidence interval
